# Interpupillary Distance and Peripapillary Myopic Changes: A Pilot Study in a Glaucomatous Cohort

**DOI:** 10.3390/jcm14144895

**Published:** 2025-07-10

**Authors:** Sameer Butt, Adèle Ehongo

**Affiliations:** Service d’Ophtalmologie, Hôpital Universitaire de Bruxelles (HUB), CUB Hôpital Erasme, Route de Lennik 808, 1070 Bruxelles, Belgium; samebutt@ulb.be

**Keywords:** myopia, interpupillary distance, peripapillary myopic changes, gamma peripapillary atrophy, ovality index, myopic complications, optical coherence tomography (OCT), axial length

## Abstract

**Background/Objectives**: Myopia is associated with peripapillary changes, namely, gamma peripapillary atrophy (γPPA) and optic disc ovalization, estimated by the ovality index (OI). These changes have been suggested to be promoted by adduction. Recent studies highlight that near reading significantly contributes to the development and progression of myopia and that the interpupillary distance (IPD) influences vergence amplitudes. While both adduction and convergence are involved during near reading, a potential link between IPD and myopic peripapillary changes has not yet been explored. We, therefore, sought to determine whether IPD is related to the OI or γPPA width. **Methods**: In this monocentric cross-sectional study, 100 eyes from 100 adults (mean age of 62.6 ± 13.7 years) were analyzed. Axial length (AL), refractive error, and IPD were recorded. The OI and γPPA width were assessed using spectral-domain Optical Coherence Tomography. Pearson correlations and multivariable linear regressions were performed, adjusting for age, gender, and myopia status. **Results**: IPD showed no significant correlation with the OI (r = 0.001; *p* = 0.989) or γPPA (r = −0.028; *p* = 0.789). A weak, non-significant correlation was found between IPD and AL (*p* = 0.059). In contrast, AL was strongly correlated with both a lower OI and wider γPPA (*p* < 0.001). **Conclusions**: These findings suggest that IPD-related biomechanical forces do not influence optic nerve head (ONH) shape or γPPA. Axial elongation remains the key driver of myopic ONH remodeling.

## 1. Introduction

Myopia is an emerging global health issue, with projections estimating that by 2050, 50% of the world’s population will be myopic and 10% will develop high myopia [1]. This growing prevalence, accentuated by behavioral changes particularly during the COVID-19 pandemic, is expected to increase visual impairment and impose significant socioeconomic burdens [2,3].

In myopia, the elongation of the eyeball induces structural eye changes, including in the peripapillary region [4]. This includes the ovalization of the optic disc [5], along with the presence and increased prominence of gamma peripapillary atrophy (γPPA), which is characterized by an atrophic region adjacent to the optic disc, most commonly located temporally in myopic eyes, and is thought to result from a mismatch between the termination of Bruch’s membrane and the optic disc opening [4,6].

**Figure 1:**
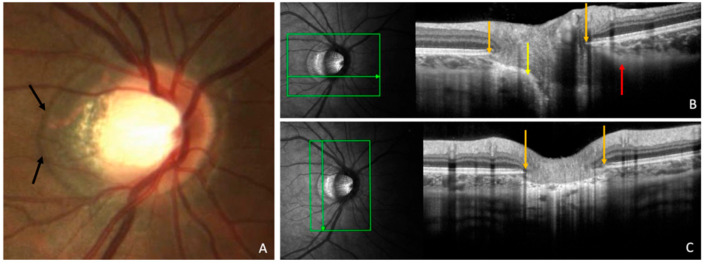
Illustration of gamma peripapillary atrophy (γPPA). (**A**) Fundus photograph of a right eye showing the extent of γPPA (black arrows). (**B**) OCT section along the horizontal green arrow in the corresponding infrared image. γPPA is between Bruch’s membrane ending (orange arrow) and the optic disc margin (yellow arrow). BMO is shifted into the temporal direction between the orange and yellow arrows; Bruch’s membrane overhangs into the intrapapillary compartment at the nasal disc border, between the orange and red arrows. (**C**) OCT section along the vertical green arrow in the corresponding infrared image showing γPPA between two orange arrows. Reproduced (with permission) from Ehongo A. Optical coherence tomography analysis of peri-papillary intrachoroidal cavitation. PhD thesis, Université libre de Bruxelles, Brussels, 2024. p. 24 [7].

As illustrated in Figure 1, γPPA represents the zone between the end of Bruch’s membrane and the optic disc border [7].

Histological and Optical Coherence Tomography (OCT) imaging studies confirm the absence of Bruch’s membrane in γPPA [8,9], which extends from the edge of Bruch’s membrane opening (BMO) to the anterior scleral opening (ASO).

**Figure 2:**
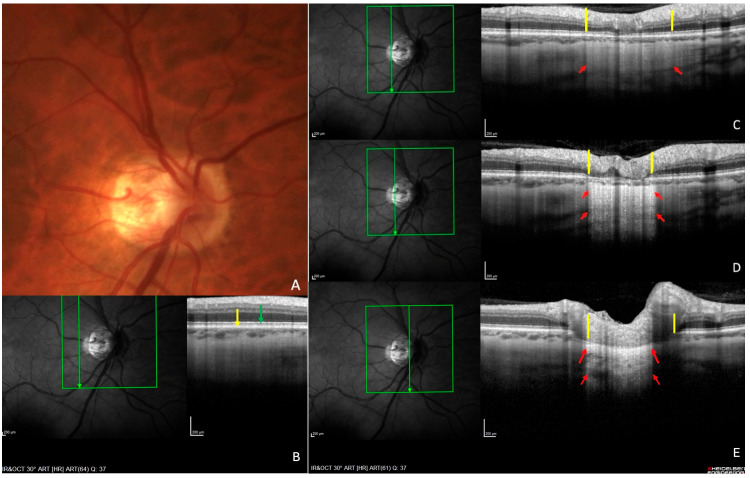
Alpha, beta, and gamma peripapillary atrophies (PPAs). (**A**) Fundus photograph of a right eye with PPA. (**B**–**E**) OCT sections along the green arrow of the corresponding infrared images. The area of PPA shows hyper-reflectivity in the infrared images. (**B**) Section outside the PPA: normal architecture of retinal pigment epithelium (RPE) (green arrow) and Bruch’s membrane (BM) (yellow arrow). (**C**) Section through alpha PPA: irregularities of the RPE are seen between the two yellow lines. (**D**) Section through beta PPA: complete absence of the RPE between the two yellow lines. The BM remains intact in both (**C**,**D**) but disappears in (**E**), corresponding to gamma PPA. As the RPE is irregular in (**C**) and absent in (**D**,**E**), more light penetrates the deeper layers, resulting in increased hyper-reflectivity (between the red arrows) compared with (**B**). Abbreviations: PPA = peripapillary atrophy. RPE = retinal pigment epithelium. BM = Bruch’s membrane. Reproduced (with permission) from Ehongo A. Optical coherence tomography analysis of peripapillary intrachoroidal cavitation. PhD thesis, Université libre de Bruxelles, Brussels, 2024. p. 26 [7].

Two other types of peripapillary atrophy (PPA) have been distinguished through histological analyses. Unlike γPPA, both retain an intact Bruch’s membrane. Alpha PPA (αPPA) is characterized by irregularities in the retinal pigment epithelium (RPE) cells, and beta PPA (ßPPA), shows a complete loss of the RPE. These changes are also well visualized with OCT [10]. Figure 2 illustrates the three main types of PPA. In highly elongated eyes, delta PPA represents a subtype of γPPA presenting the thinning of the peripapillary scleral flange [10].

The degree of optic disc deformation is approached by the ovality index (OI), calculated as the ratio of the shortest to the longest disc diameters [11]. Both the OI and γPPA are proposed to quantify myopic structural peripapillary changes.

A recent biomechanical hypothesis suggests that the traction force exerted by the optic nerve sheaths (ONSs) may contribute to axial elongation in myopia [12]. This hypothesis draws on prior research demonstrating that the ONSs’ traction force aligns with the magnitude of forces generated by the extraocular muscles and acts in the axial direction. Such traction is proposed to induce significant peripapillary forces, particularly during adduction movements exceeding 26 degrees [13].

Such forces have been implicated in a spectrum of clinically relevant peripapillary myopic complications, in particular visual impairment or associated visual field defects mimicking those of glaucomatous optic neuropathy [14,15,16,17]. However, the factors that determine these peripapillary complications remain to be clarified.

Importantly, recent studies have highlighted that near-reading activities and increased digital screen time are particularly associated with greater myopia progression [18]. During such near-reading activity, the eyes not only adduct but also converge.

Interestingly, interpupillary distance (IPD) has been shown to influence vergence amplitudes [19]. Although near activities involve both adduction and convergence, to our knowledge, no study has examined whether IPD might be linked with structural peripapillary changes related to myopia through its role in vergence mechanisms.

Therefore, we aimed to investigate a potential correlation between IPD and two quantitative markers of structural peripapillary changes in myopic eyes: the OI and γPPA.

## 2. Materials and Methods

This was an observational, cross-sectional, monocentric study approved by the Ethics Committee of Erasmus Hospital (Brussels, Belgium) (reference P2024/450) and the Institutional Review Board (reference SRB2024239). This study complied with the tenets of the Declaration of Helsinki, and written informed consent was acquired from all participants.

### 2.1. Inclusion Population

Participants were approached during their routine visits to our glaucoma outpatient clinic and were consecutively recruited over a two-month period (January–February 2025). A target of at least 50 myopic eyes was set, given that this is a pilot study and the correlation between IPD and both the OI and γPPA has never been studied before.

### 2.2. Inclusion Criteria

Participants aged 18 years or older with no history of strabismus, strabismus surgery, posterior segment surgery, or trabeculectomy were included. An OCT signal quality score of at least 25 was also required.

### 2.3. Exclusion Criteria

Participants were excluded if they were younger than 18 years, had a history of ocular trauma, glaucoma surgery, posterior segment surgery, or strabismus surgery. Eyes with optic nerve anomalies unrelated to myopia or glaucoma or with an OCT signal quality score below 25 were also excluded.

### 2.4. Myopic Group

The classification of myopia was primarily based on the spherical equivalent (SE ≤ −0.50 D), in accordance with the International Myopia Institute [20]. In cases where refractive error (RE) data were missing or deemed unreliable, axial length (AL) was used as a secondary criterion. Specifically, if AL was ≥26.0 mm, the eye was classified as myopic. If AL was <26.0 mm, the eye was classified as “undefined” with respect to RE. This approach enabled classification even in the absence of reliable refractive data, as in the following scenarios: (a) eyes with a history of refractive surgery, for which preoperative refraction was not available for analysis; (b) eyes with a history of cataract surgery without available preoperative remote refraction from cataract documentation; and (c) eyes with unoperated but clinically significant cataract at the time of inclusion, for which no reliable pre-cataract refraction could be retrieved.

This strategy was also chosen to ensure that eyes with moderate or low myopia were not misclassified as non-myopic due to shorter axial lengths. Relying solely on axial length could have led to the underrepresentation of these patients, especially since axial elongation does not always correlate linearly with refractive error [20]. Therefore, the spherical equivalent was prioritized when available, as it better captures the full range of myopia severity.

The use of a 26.0 mm threshold aligns with the established definition of high myopia as a subgroup of eyes with a high degree of myopic refractive error. High myopia is distinct from pathologic myopia, which is defined by complications of myopia that may also occur in eyes without high myopia [21].

### 2.5. General and Ocular Data

Age and gender were recorded for each subject. Data from the comprehensive ophthalmic examination were retrieved for both eyes of the subjects. For each eye, the following parameters were recorded: RE in SE, AL values using IOL Master^®^ (Carl Zeiss Meditec, Jena, Germany), and IPD measurements with auto-refraction (TONOREF II, model RKT-2014; NIDEK Co., Ltd., Tokyo, Japan). Color fundus pictures using a Clarus^®^ non-mydriatic cameras (PRO NM Carl Zeiss Meditec, Jena, Germany) and spectral-domain OCT imaging using a Spectralis^®^ S3300 model, version 6.16.2 (Heidelberg Engineering GmbH, Heidelberg, Germany), were analyzed.

### 2.6. Color Fundus Photographs

These images were used to exclude eyes with retinal or optic nerve anomalies unrelated to myopia or glaucoma, in order to eliminate potential confounding factors in the interpretation of structural findings.

### 2.7. Eye Selection

Although a full ophthalmic examination was available for both eyes of each participant, only the right eye was included in the study unless it met an exclusion criterion, in which case the left eye was used instead. This approach was adopted to account for inter-eye correlation.

### 2.8. OCT Imaging

Radial OCT section acquisition using the commercialized Glaucoma Premium Edition module of the Spectralis^®^ performed for routine glaucoma documentation was used, specifically the 48 radial line B-scans centered on the optic disc of each participant for the tomographic analyses of the peripapillary region.

### 2.9. OCT Analysis

OCT sections and infrared images were opened in display mode; then, γPPA width and the OI were measured.

- Measurement of width of γPPA

**Figure 3:**
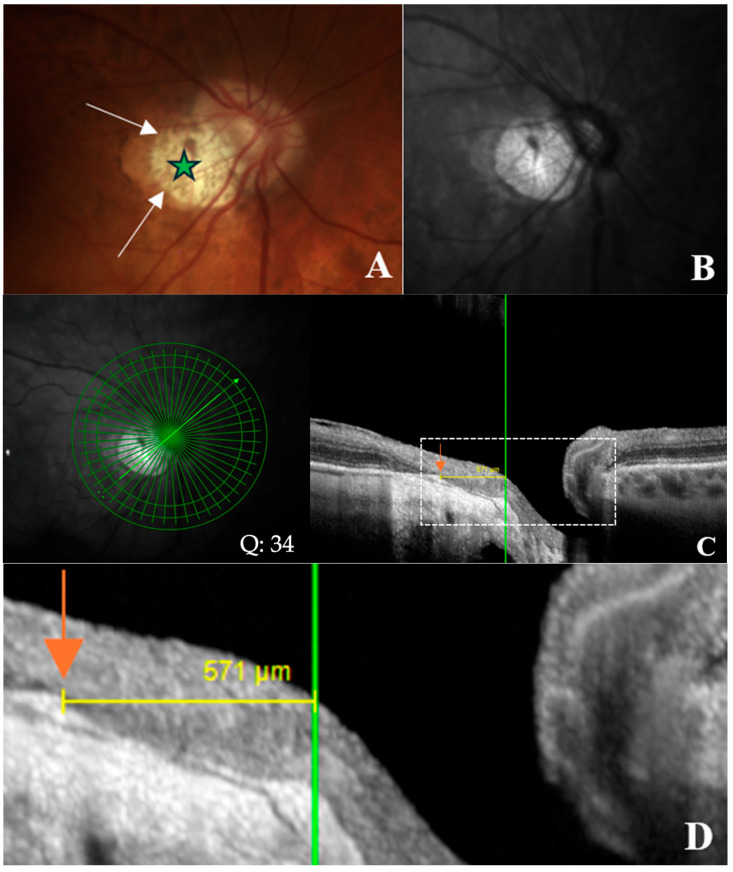
OCT-based measurement of γ-zone peripapillary atrophy (γPPA) width. (**A**) Fundus image illustrating end of γPPA (white arrows) and temporal myopic conus (green star). (**B**) Infrared image without the radial line scan tool. (**C**) Section along the green arrow in the infrared image. The γPPA is visualized as the atrophic zone between Bruch’s membrane opening (BMO; orange arrow) and the anterior scleral opening (ASO; green line). The yellow arrows with calipers indicate the γPPA width measured along the short axis. Signal quality score: Q = 34. (**D**) A magnified view of the white dashed rectangle in (**C**) is provided to improve the readability of the measurements. In this case, the γPPA measures 571 µm.

The width of γPPA, which is the PPA without Bruch’s membrane [8,9], was assessed using radial OCT. This atrophic zone extends from the BMO to the ASO (Figure 3A–D). The measurement of the γPPA width was performed along the minor axis, from the BMO indicated by the orange arrow, to the ASO, indicated by the vertical green line (Figure 3C,D).

- OI measurement

**Figure 4:**
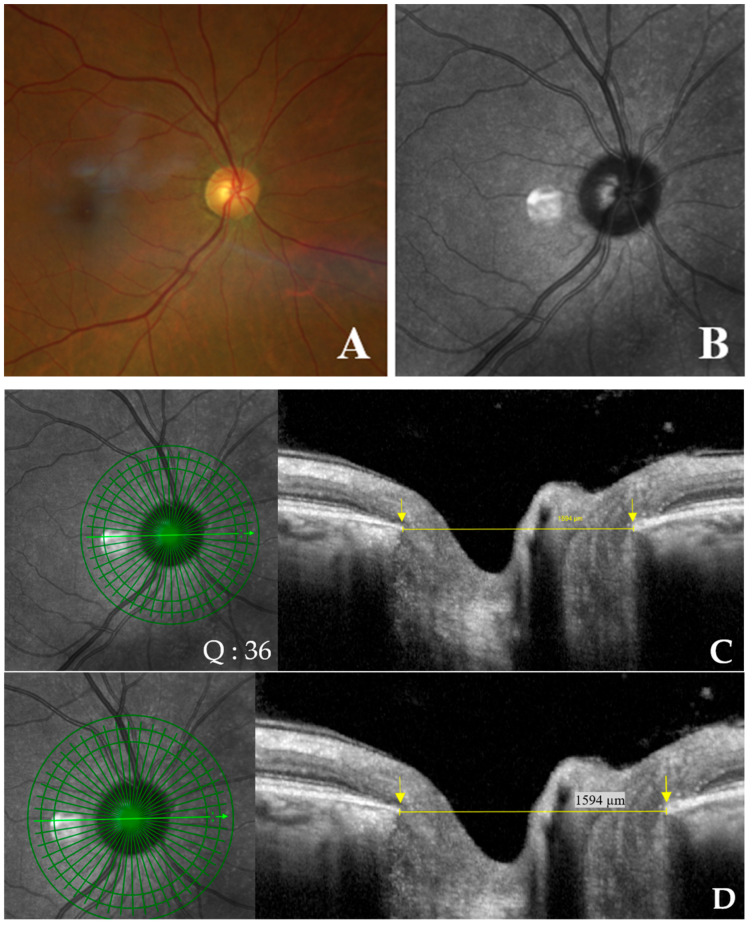
Schematic representation of optic disc measurements in an eye without gamma peripapillary atrophy (γPPA). (**A**) Normal fundus image of right eye. (**B**) Infrared image without the radial line scan tool. (**C**) Section along the green arrow in the infrared image. Yellow arrow calipers indicating the measurement between temporal Bruch’s membrane opening (BMO) and nasal BMO (1594 μm). Arrows indicate BMO (yellow). Signal quality score: Q = 36. (**D**) Enlarged view from (C), showing the same structures with improved readability of the measurements.

The degree of disc deformation was quantified using the OI, calculated as the ratio of the shortest (minor axis) to longest (major axis) disc diameters [11] (Figure 4A–D). In the absence of γPPA, the diameter was measured from the BMO to the BMO [22] (Figure 4C,D).

**Figure 5:**
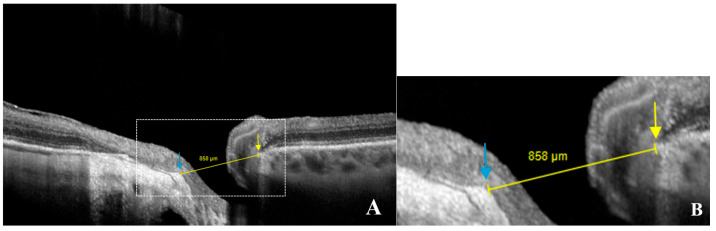
Schematic representation of optic disc measurements in an eye with gamma peripapillary atrophy (γPPA). (**A**) Yellow arrow calipers indicating the measurement between the anterior scleral opening (ASO) (temporal) and nasal Bruch’s membrane opening (BMO) (858 μm). Arrows indicate ASO (blue) and BMO (yellow). (**B**) Magnified view of the region within the white dashed rectangle, showing the same structures in greater detail to improve the readability of the measurements. (Same patient as in Figure 3).

In the presence of γPPA resulting from the temporal shifting of the BMO relative to the ASO, the diameter was measured from the ASO (temporal) to the BMO (nasal) (Figure 5A,B).

**Figure 6:**
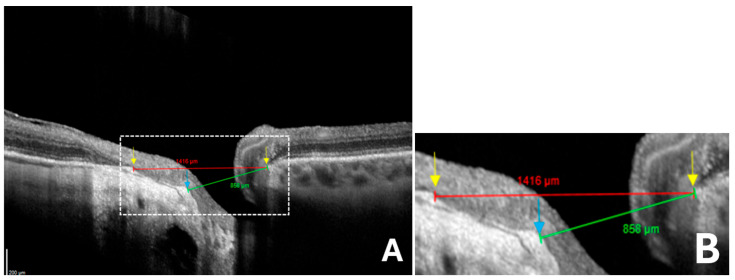
Comparison between standard and anatomically adjusted optic disc diameters measurements for ovality index calculation in eyes with gamma peripapillary atrophy (γPPA). (**A**) Red arrow calipers indicate measurement of the disc diameter, taken between temporal Bruch’s membrane opening (BMO) and nasal BMO (1416 μm). This measurement is overestimated due to the temporal shifting in Bruch’s membrane. Green arrow calipers show the neural canal opening measurement between the temporal anterior scleral opening (ASO) and the nasal BMO (858 μm), corresponding to the anatomical landmarks used for disc ovality index measurement. Arrows indicate ASO (blue) and BMO (yellow). (**B**) Magnified view of the region within the white dashed rectangle, showing the same structures in greater detail to improve the readability of the measurements. (Same patient as in Figure 3).

A standard minor axis measurement between BMO (nasal) and BMO (temporal) would overestimate its length, leading to an artificially high OI (Figure 6).

### 2.10. Analysis Procedure

OCT images from all eyes were analyzed by the investigator (Butt Sameer, B.S.), who performed the measurements. These measurements were subsequently reviewed and verified by the principal investigator, an experienced ophthalmologist (Ehongo Adèle, E.A.), to ensure accuracy.

### 2.11. Statistical Analyses

Descriptive statistics were presented as means, standard deviations (SDs), and ranges for continuous variables and as proportions and percentages for categorical variables. The Mann–Whitney U test was used to compare continuous variables between two groups. Due to the small sample sizes resulting from subgroup divisions, non-parametric tests were preferred. Statistical analyses were performed using IBM SPSS Statistics version 30.0. Pearson correlation analyses and multivariable regression models were conducted. The three outcome variables, namely, γPPA, OI, and AL, were analyzed separately as dependent variables. IPD was used as the main independent variable in all models, which were adjusted for age, gender, and myopia. Finally, a binary variable indicating the presence or absence of γPPA was included as a control variable in regression models where γPPA width was the dependent variable. A *p*-value < 0.05 was considered statistically significant.

## 3. Results

### 3.1. Sample Characteristics

Overall, 100 eyes of 100 subjects, 60 of whom were females, were included. The sample comprised 96 right eyes and 4 left eyes. The mean age ± SD was 62.6 ± 13.7 years, within a range of (20–83). The demographic and ocular features of the sample population are summarized in Table 1. A total of 83 eyes had reliable RE data.

Of the 17 eyes with unreliable refractive error, 10 eyes had a history of phacoemulsification with unavailable remote pre-cataract refractive data. Similarly, refractive data were excluded from five eyes with unoperated but clinically significant cataract, for which no prior refraction was available. Finally, two eyes with a history of refractive surgery were identified, and their refractive data were excluded, as no preoperative refraction was available.

Among the 17 eyes without reliable RE and excluded for refractive data, 7 were classified as myopic based on AL ≥ 26.0 mm, and 10 could not be classified due to AL < 26.0 mm. Ultimately, 52 eyes were classified as myopic and 38 as non-myopic, and 10 remained unclassified (Table 2).

The mean age and IPD were not significantly different between myopic and non-myopic groups. In contrast, all other parameters showed statistically significant differences. Participants with unclassified myopic status were significantly older and had a significantly shorter AL compared with those in the myopic group (Table 2).

Among the 100 eyes included in the study, 35 presented with γPPA. There was no statistically significant difference in mean age and IPD between participants with and without γPPA. In contrast, all other measured parameters demonstrated statistically significant differences between the two groups (Table 3).

### 3.2. Correlation Analyses

Pearson correlation analyses revealed no significant correlation between IPD and the OI (r = 0.001, *p* = 0.989), nor between IPD and γPPA width (r = −0.028, *p* = 0.782).

**Figure 7:**
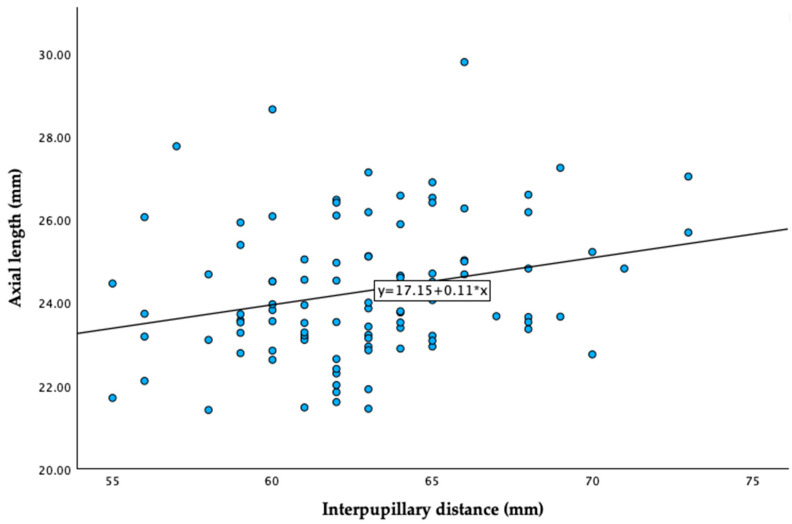
Correlation between interpupillary distance and axial length. Equation of the regression line: Axial length (mm) = 17.15 + 0.11 X interpupillary distance (mm). Pearson coefficient = 0.256; *p* = 0.011.

A significant but weak positive correlation was found between IPD and AL (r = 0.256, *p* = 0.011) (Table 4, Figure 7). Additionally, a significant correlation was observed between γPPA width and the OI, and both parameters showed significant correlations with AL (Table 4).

### 3.3. Multivariable Regression Analyses

Multivariable linear regression models were performed, including age, gender, myopia, and the presence or the absence of γPPA as control variables.

No significant association was found between IPD and γPPA width after adjustment (β = −3.814; 95% CI: −13.67 to 6.04; *p* = 0.44). Similarly, no significant association was observed between IPD and the OI (β = −0.001; 95% CI: −0.006 to 0.005; *p* = 0.85). A marginal trend toward significance was noted between IPD and AL (β = 0.078; 95% CI: −0.003 to 0.160; *p* = 0.059).

### 3.4. Sensitivity Analyses

Complementary analyses were conducted, including a logarithmic transformation of γPPA and Spearman correlation testing, both of which yielded similar results. These additional analyses are presented in Appendix A.

## 4. Discussion

Structural changes in the ONH and peripapillary region in myopic eyes have become a major focus of current research [23], given their clinical relevance, particularly their potential to mimic or overlap with glaucomatous visual field defects, including those seen in normal-tension glaucoma [14,17]. To our knowledge, this is the first study to investigate the relationship between IPD and myopic peripapillary changes, namely, the OI and γPPA width. Our findings showed no significant correlation between them, suggesting that IPD-related adduction forces may not play a major role in shaping ONH morphology in myopic eyes.

In contrast, we observed significant correlations between AL and both the OI and γPPA width. These findings support the established role of axial elongation as a major driver of myopic structural changes [4,24,25]. Moreover, the strong correlation between the OI and γPPA suggests that the change in optic disc shape and peripapillary changes are closely related, likely due to a shared underlying mechanism. Previous studies have shown that these deformations develop simultaneously during ocular elongation [4,24]. It is important to emphasize that myopic complications result from several structural deformations that develop simultaneously as the eye elongates. Changes such as optic disc obliquity and γPPA often occur together and may interact with other anatomical alterations. This cumulative remodeling process likely underlies more complex forms of myopic damage, including peripapillary intrachoroidal cavitation [17,26].

Structurally, myopic eyes have a longer AL. This increased AL compromises the sclera’s structure, reduces its rigidity and changes its biomechanical properties, making it more susceptible to deformation [27]. In eyes with marked AL, the traction on the optic disc may result from a short ONS or reduced sheath elasticity, both of which could restrict full ocular adduction [28].

Despite the weak correlation observed between IPD and AL (r = 0.256, *p* = 0.011), potentially reflecting shared developmental pathways influencing craniofacial and ocular growth [29], this relationship did not translate into the OI and γPPA after adjusting for confounding factors. After adjusting for AL, age, and gender, the correlation between IPD and the OI (β = −0.001, *p* = 0.85), as well as that between IPD and γPPA (β = −3.814, *p* = 0.44), remained statistically non-significant. These results suggest that axial elongation exerts a predominant influence, potentially overshadowing any biomechanical effects associated with IPD.

Although the subjects in this study were recruited from a glaucoma clinic, the optic disc changes underpinning the diagnosis of glaucomatous optic neuropathy (GON) are well established and follow a characteristic pattern, primarily involving loss of the neuroretinal rim, the hallmark of GON [10]. It is important to note that our study did not aim to assess the neuroretinal rim. Instead, we focused on γPPA (the PPA lacking Bruch’s membrane) and on the OI. To estimate the OI, Bruch’s membrane opening as the optic nerve border was considered. Unlike αPPA, which is present in almost all eyes [30], and ßPPA, which tends to enlarge with glaucoma progression, both of which were beyond the scope of this study, γPPA is associated with axial elongation in myopia. Therefore, GON-related changes do not represent a confounding factor in our analysis of γPPA. Moreover, the distinct histological characteristics of each PPA subtype can be clinically distinguished by OCT imaging [10], reducing the risk of misinterpretation.

Sclerosis of the lens may be a potential confounding factor affecting the refractive error data in this study. However, it is noteworthy that the mean age of the myopic group was slightly lower than that of the non-myopic group (although this difference did not reach statistical significance), suggesting that lens sclerosis is unlikely to be the determining factor in the negative refractive values observed in the myopic group.

The advanced mean age of our cohort (62.6 ± 13.7 years) was considered a potential confounding factor in the analysis of γPPA, given the positive correlation between age and cumulative myopic exposure, due to the progressive nature of axial elongation over time [31]. Accordingly, γPPA analyses were adjusted for age using linear regression. Interestingly, age did not differ significantly between myopic and non-myopic groups. Similarly, no significant age difference was observed between eyes with and without γPPA.

Overall, adjustments were made for the parameters age, gender, and myopia to account for potential confounding factors. Age was included because peripapillary changes naturally worsen with aging, independent of myopia [32]. Gender was controlled for, as women generally exhibit a smaller IPD compared with men, and this anatomical difference could indirectly influence outcomes [33]. Myopia was adjusted for, as its severity (assessed via axial elongation) is a major driver of ONH deformations [34].

Regarding the sample size, it is important to note that this study aimed to include eyes exhibiting both γPPA and an oval disc configuration. Since these changes are commonly associated with myopia, we initially planned to recruit 50 myopic eyes and a similar number of non-myopic eyes as controls. Ultimately, we included 52 myopic eyes and 35 eyes presenting γPPA. As a pilot study, these findings provide useful data for refining sample size calculations in future research, helping to address the sample size limitations of the present work.

Retinal image magnification varies with ocular biometry, so lateral (retina-parallel) measurements obtained on OCT are inherently affected [35,36]. The Spectralis^®^ OCT platform mitigates this automatically: its HEYEX software (v6.16.2), built on the Gullstrand schematic eye, applies an individualized lateral scaling factor based on each eye’s biometry [37,38]. Even so, some investigators propose adding an extra correction for AL, while others contend that entering a patient’s mean keratometry during scan acquisition offers a more accurate adjustment than the default setting [38]. Conversely, very recent work by Kirik et al. indicates that with Spectralis^®^ OCT, further AL compensation is unnecessary [39]. Therefore, we did not perform any corrections.

Myopia is of concern to most ophthalmic subspecialties due to its increasing prevalence, early onset, and diverse complications. We briefly reviewed its classification according to RE and AL in the Materials and Methods Section [20]. AL allows for a practical division of the myopic population into two subgroups: high myopes and non-high myopes. However, although pathological myopia (defined as myopia with ocular complications) is frequently associated with high myopia, such complications, including posterior staphyloma, may also occur in eyes with a normal AL [21]. These observations suggest that axial length alone may not be sufficient to identify myopes at risk of complications. In the future, it would be interesting to compare groups with and without high myopia, within the broader myopic population, in terms of IPD. A significant difference in IPD between these groups could help identify subgroups of myopic individuals at higher risk of developing high and/or pathological myopia.

Despite advances in our understanding of myopia-related complications [10,40], many questions remain unanswered, which justifies the need for continued research. Near work has been consistently associated with both the onset and progression of myopia. Demonstrating a potential link between IPD and structural markers such as the OI or γPPA could open avenues for new preventive strategies. One such approach might involve the use of prisms in reading glasses to reduce the convergence demand, thereby limiting myopia progression. Further studies addressing the limitations of the present work are warranted.

### Limitations

First, this was a cross-sectional pilot study. This design limitation warrants future confirmatory and longitudinal investigations.

Second, differences in nasal bridge anatomy may influence the extent of the visual field. Specifically, flatter nasal bridges have been shown to allow for greater visibility in the nasal field during extreme gaze, due to reduced occlusion [41]. This anatomical variation may partly explain population-level differences in ONH changes associated with myopia. In our study, the nasal bridge structure was not assessed. Yet, for comparable IPD, differences in nasal anatomy, such as flatter bridges, may permit greater adduction, potentially contributing to the observed disparities in myopic ONH changes across populations. Including direct measurements of nasal morphology or stratifying analyses by ethnicity would have strengthened the interpretation of our findings.

Third, it is plausible that individuals with larger IPD require greater ocular movement amplitudes to maintain binocular vision, which could theoretically impact the binocular visual field. However, this adaptation largely depends on individual’s vergence capacity. Dag et al. have suggested that individuals with larger IPD may exhibit reduced vergence amplitudes, potentially indicating a less stable binocular fusion [19]. While findings remain inconclusive, this raises the possibility that structural variations such as IPD might influence binocular visual performance. As we did not assess binocular visual fields in this study, we cannot determine whether differences in IPD, γPPA width, or the OI translate into functional differences in binocular vision. Future studies should include binocular visual field testing to explore these potential correlations.

Fourth, measurements were performed by a single investigator and reviewed by a senior ophthalmologist. Independent double grading should be implemented in future studies.

Fifth, the study cohort was ethnically homogeneous, composed almost exclusively of individuals of Caucasian descent, with negligible representation of other populations. This lack of diversity may limit the generalizability of our findings to other ethnic populations, where myopic ONH changes are not only more prevalent but also tend to be more severe [42]. This limitation was not accounted for in the study design and should be addressed in future studies through more inclusive sampling or stratified analyses.

Sixth, as this was a pilot study, we arbitrarily enrolled 100 eyes, 52 of which were myopic. A larger sample may have allowed for the detection of significant correlations. Therefore, future research may build upon these preliminary findings to better define sampling needs in myopic populations.

Seventh, participants were recruited from a tertiary care clinic, involving a relatively homogeneous group that may not be representative of the general population.

## 5. Conclusions

This study is the first to investigate the relationship between IPD and structural changes in the peripapillary zone in myopia, specifically focusing on the OI and γPPA. Although previous studies have suggested that IPD may influence vergence amplitude and that duction movements could promote optic nerve deformations, no significant correlation was found between IPD and either the OI or γPPA width after adjusting for confounding variables. These findings highlight the dominant role of axial elongation in driving myopic ONH and peripapillary reshaping, outweighing any potential biomechanical influence of IPD. Future studies should consider longitudinal designs, include more ethnically diverse populations, and assess the binocular visual field extent to further elucidate the complex interplay of ocular structure, craniofacial features, and visual biomechanics.

## Figures and Tables

**Table 1:** Demographic and ocular characteristics of the study sample: 100 eyes of 100 subjects.

Parameter	Sample Size (*n*)	Mean ± SD	Range
Age (years)	100	62.6 ± 13.7	20–83
Refractive error (diopter)	83	−1.54 ± 3.5	−17.50 to +3.75
Axial length (mm)	98	24.27 ± 1.6	21.42–29.81
Gamma peripapillary atrophy (µm)	100	124.27 ± 224.4	0–1183
Ovality index	98	0.88 ± 0.09	0.38–0.99
Interpupillary distance (mm)	100	62.7 ± 3.7	55–73

**Table 2:** Demographic and Ocular Characteristics by Refractive Status.

Parameter	Myopic			Non-Myopic			Unclassified				
	*n*	Mean ± SD	Range	*n*	Mean ± SD	Range	*n*	Mean ± SD	Range	P_1_	P_2_
Age (years)	52	59.06 ± 15.41	20–83	38	65.03 ± 10.66	42–83	10	72 ± 8.74	59–83	0.078	**0.009**
Refractive error (Diopter)	45	−4.03 ± 2.89	−17.5 to −0.5	38	1.4 ± 1.13	−0.37 to 3.75	0	/	/	**<0.001**	/
Axial length (mm)	52	25.26 ± 1.55	21.48–29.81	38	23.21 ± 0.83	21.42–25.12	8	22.89 ± 1.16	21.45–24.69	**<0.001**	**<0.001**
Gamma peripapillary atrophy (µm)	52	215.31 ± 269.47	0–1183	38	11.50 ± 50.09	0–253	10	79.40 ± 168.39	0–436	**<0.001**	0.066
Ovality index	50	0.85 ± 0.11	0.38–0.99	38	0.91 ± 0.05	0.77–0.99	10	0.89 ± 0.08	0.70–0.99	**0.006**	0.345
Interpupillary distance (mm)	52	63.38 ± 4.02	55–73	38	62.11 ± 3.56	55–70	10	62.10 ± 2.23	58–65	0.133	0.351

Notes: Bolded values indicate statistically significant differences for pairwise comparison. P_1_: significance level for comparisons between myopic and non-myopic groups; P_2_: significance level for comparisons between myopic and unclassified groups (Mann–Whitney U test in both cases). Abbreviation: *n*, number of eyes.

**Table 3:** Demographic and Ocular Characteristics according to the Presence or Absence of Gamma Peripapillary Atrophy (γPPA).

Parameter	γPPA Width > 0 µm *n* = 35			γPPA Width = 0 µm *n* = 65			
	*n*	Mean ± SD	Range	*n*	Mean ± SD	Range	*p*
Age (years)	35	62.26 ± 16.80	23–83	65	62.82 ± 11.97	20–81	0.696
Refractive error (Diopter)	29	−4.45 ± 3.35	−17.5 to 0.5	54	0.02 ± 2.50	−6.5 to 3.75	**<0.001**
Axial length (mm)	34	25.44 ± 1.57	21.45–29.81	64	23.65 ± 1.34	21.42–27.26	**<0.001**
Gamma peripapillary atrophy (µm)	35	355.06 ± 249.57	453–1183	65	0 ± 0	0–0	**<0.001**
Ovality index	33	0.82 ± 0.12	0.38–0.99	65	0.91 ± 0.05	0.77–0.99	**<0.001**
Interpupillary distance (mm)	35	62.94 ± 3.75	55–73	65	62.68 ± 3.75	55–73	0.604

Notes: Bold values indicate statistically significantly differences between γPPA > 0 and γPPA = 0. *p*: significance of mean differences between γPPA > 0 and γPPA = 0 groups, Mann–Whitney U test. Abbreviations: γPPA, gamma peripapillary atrophy; *n*, number of eyes.

**Table 4:** Pearson correlation matrix among ocular parameters.

		γPPA	OI	IPD	AL
γPPA	Pearson Correlation	1	−0.694 **	−0.028	0.547 **
	*p*-Value		<0.001	0.782	<0.001
OI	Pearson Correlation	−0.694 **	1	0.001	−0.417 **
	*p*-Value	<0.001		0.989	<0.001
IPD	Pearson Correlation	−0.028	0.001	1	0.256 *
	*p*-Value	0.782	0.989		0.011
AL	Pearson Correlation	0.547 **	−0.417 **	0.256 *	1
	*p*-Value	<0.001	<0.001	0.011	

**. Correlation is significant at the 0.01 level (2-tailed). *. Correlation is significant at the 0.05 level (2-tailed). Abbreviations: γPPA, gamma peripapillary atrophy; OI, ovality index; IPD, interpupillary distance; AL, axial length.

**Figure A1:**
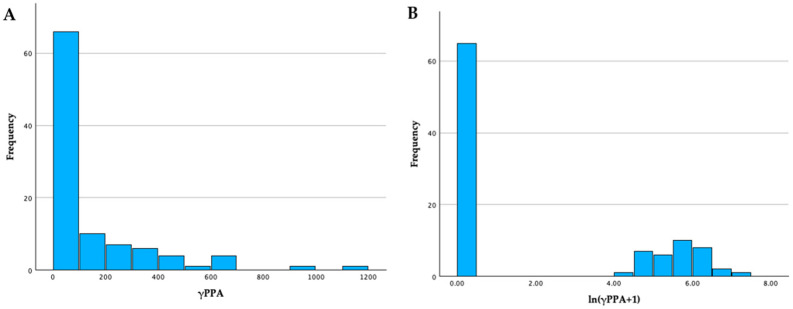
Distribution of gamma peripapillary atrophy (γPPA) before and after logarithmic transformation. (**A**) Histogram A: Original distribution of γPPA showing strong a right skew and a high frequency of zero values. (**B**) Histogram B: The log-transformed variable ln (γPPA + 1) shows improved normality.

## Data Availability

The data presented in this study are available upon request from the corresponding author due to privacy and ethical restrictions.

## Abbreviations

The following abbreviations are used in this manuscript:

AL	axial length
ASO	anterior scleral opening
BMO	Bruch’s membrane opening
GON	glaucomatous optic neuropathy
IPD	interpupillary distance
OCT	Optical Coherence Tomography
OI	ovality index
ONH	optic nerve head
ONS	optic nerve sheath
PPA	peripapillary atrophy
αPPA	alpha peripapillary atrophy
ßPPA	beta peripapillary atrophy
γPPA	gamma peripapillary atrophy
RE	refractive error
RPE	retinal pigment epithelium
SD	standard deviation
SE	spherical equivalent

## Appendix A

### Appendix A.1. Sensitivity Analyses (Details)

Given the right-skewed distribution of γPPA width and the substantial number of zero values, a logarithmic transformation ln (γPPA + 1) was explored in an attempt to improve data normality (Figure A1). Furthermore, a binary variable (γPPA_dummy) was created to distinguish between the absence and presence of γPPA.

However, this transformation yielded comparable overall results (Table A1). Therefore, analyses were ultimately conducted using the original (untransformed) γPPA width values.

**Table A1:** Adjusted multivariable regression models assessing the association between IPD and γPPA, in both original and log-transformed forms.

Dependent Variable	Predictors Included	β (95% CI) for IPD	*p*-Value
γPPA	IPD, age, gender, myopia, and γPPA_dummy	−3.814 (−13.67; 6.05)	0.444
ln (γPPA + 1)	IPD, age, gender, myopia, and γPPA_dummy	−0.016 (−0.041; 0.010)	0.231

Note: Each model was adjusted for age, gender, γPPA_dummy, and myopia. The table shows the effect of interpupillary distance (IPD) on γPPA in its original and log-transformed form. Only the coefficient for IPD is reported. Abbreviations: γPPA, gamma peripapillary atrophy; IPD, interpupillary distance. N.B.: γPPA_dummy (γPPA absent = 0; γPPA present = 1).

In addition, due to the non-optimal normality of certain continuous variables, non-parametric Spearman correlation analyses were also performed alongside Pearson correlations (Table A2). Since the results were comparable across both methods, only Pearson correlation coefficients are reported in the main text.

**Table A2:** Pearson vs. Spearman correlation coefficients.

Variable Pair	r (Pearson)	*p*-Value (Pearson)	ρ (Spearman)	*p*-Value (Spearman)
IPD-γPPA	−0.028	0.782	0.028	0.785
IPD-OI	0.001	0.989	−0.006	0.949
IPD-AL	0.256 *	0.011	0.274 **	0.006
γPPA-OI	−0.694 **	<0.001	−0.403 **	<0.001
γPPA-AL	0.547 **	<0.001	0.554 **	<0.001
AL-OI	−0.417 **	<0.001	−0.275 **	0.007

Note: **: Correlation is significant at the 0.01 level (2-tailed); *: Correlation is significant at the 0.05 level (2-tailed). Abbreviations: γPPA, gamma peripapillary atrophy; OI, ovality index; IPD, interpupillary distance; AL, axial length.

These additional exploratory analyses were conducted to ensure the robustness and reliability of the main statistical results presented.
